# Editorial: Nicotine and its derivatives in disorders of cognition: a challenging new topic of study

**DOI:** 10.3389/fnins.2023.1252705

**Published:** 2023-07-18

**Authors:** Albert Gjedde

**Affiliations:** Department of Neuroscience, Faculty of Health Sciences, University of Copenhagen, Copenhagen, Denmark

**Keywords:** nicotine, cotinine, smoking, aging, juveniles

Nicotine is a compound of considerable interest to neuroscience, in contexts of physiology as well as pathology of brain functions related to neurotransmitter mechanisms. Nicotine is an alkaloid that exists naturally in plants such as tomatoes and potatoes, with the highest levels in the tobacco plant. The plants belong to the nightshade family of *Solanaceae*, including the black nightshade members that tend to be poisonous. In mammalian brains, nicotine has multiple actions that appear to be accidents of evolution, as no specific relation springs to mind between the functions of nicotine in plants and animals. The following discussion expands upon the three topics of biology, therapy, and possible prevention, as related to cognition, in the three reviews and the three original studies included in the collection.

## Biology

Conley et al. completed a pilot study of transdermal application of nicotine to patients with late-life depression (LLD) to test the hypothesis that nicotine would improve the cognitive performance and mood of the patients. The LLD condition is associated with deficits of cognitive performance combined with poor response to antidepressant medication. The investigators used electroencephalography (EEG) to test the hypothetical mechanisms that could underlie an effect of nicotine. Nicotine augmentation appeared to improve the performance on an auditory oddball task, and the analysis of event-related oscillations showed that nicotine treatment coincided with reduced beta desynchronization and improved mood symptoms. The study provides further evidence for the impact of nicotine on cortical activity and mood in depressed older adults. It also shows the utility of EEG as a marker of nicotinic mechanisms in geriatric patients. In this sense, we may view the study of Conley et al. as a follow-up to the systematic review and meta-analysis by Majdi et al. (2021) of the effects of nicotine on cognitive functions in healthy non-smoking adults. In this meta-analysis, the effects of nicotine patches in healthy non-smoking adults showed that transdermal nicotine has significant positive effects on attention but no effects on memory. Conti and Baldacchino compared the origins of nicotine's effects in early- and late-onset smokers, using voxel-based morphometry to compare gray- and white-matter volume differences in key brain regions of the frontal cortex and striatum. The pathological effects of smoking on brain physiology and brain functions are well-known, as are the ability of smoking to reverse the pathological reductions that are evident when smokers briefly discontinue the habit (Vafaee et al., 2015). The effects of smoking raise the question of the specific effects of the nicotine component of tobacco smoke. Cumming et al. (1999) discovered that [^11^C]-(S)-nicotine, a ligand for nicotinic receptors, undergoes a rapid transformation to [^11^C]-(S)-cotenine that is less polar than the parent compound. In the subsequent experiments of Cumming et al. (2003), a rather high dose of nicotine acutely reduced the binding of the dopamine receptor ligand [^11^C]raclopride by 10% in ventral striatum, with effects later moving to caudate and dorsal putamen. The magnitude of the procognitive effects of nicotine remain controversial because of possible effects of pharmacologically active metabolites of nicotine on mechanisms of learning and memory. Majdi et al. (2019) subsequently reported evidence from the literature of effects of nicotine metabolites on cognition that points to three pharmacologically active metabolites of nicotine in the brain, of which **cotinine** significantly improved cognition with no adverse effects.

## Therapy

In an earlier review, Majdi et al. (2018) tested the claim that nicotine attenuates the signs of brain dysfunction in a model of brain aging of mice induced by D-galactose (DGal). The authors tested nicotine at different doses by subcutaneous or intranasal administration to mice that received DGal for 6 weeks. The nicotine administration significantly improved spatial and episodic memories and reduced mitochondrial damage. The authors concluded that nicotine can attenuate age-related cognitive impairment at doses that raise neurotrophic factors without withdrawal signs. The question is of the specific agent or agents responsible for this effect, if any. In their review, Echeverria et al. present cotinine as the cholinergic modulator with neuroprotective, antidepressant, anti-inflammatory, antioxidant, and memory-enhancing effects, based on recent evidence suggesting that cotinine's beneficial effects result from modulation of α7 nicotinic acetylcholine receptors (nAChR) and inhibition of toll-like receptors (TLR). The α7 nAChR appear to affect brain functions by modulating inhibitory and excitatory neurotransmission mediated by GABA interneurons. Cotinine's interaction with the α7 nAChR and TLR appears to reduce neuroinflammation by inhibiting the release of pro-inflammatory cytokines by the immune cells. Kim et al. tested “alternative” effects of yoga and aerobic exercise on response inhibition and possible underlying neural mechanisms in adult smokers, along with changes of craving and affect. The participants completed experimental sessions of yoga and aerobic exercise, and both yoga and aerobic exercise significantly reduced negative affect, while craving only declined after yoga. Event-related potentials showed that P3 amplitudes declined more after yoga than after aerobic exercise, suggesting increased neural efficiency after yoga, with reduced neural activity with the same cognitive performance as aerobic exercise. The authors claim that video-based yoga practice may provide additional benefits, potentially reaching many smokers in a non-face-to-face manner.

## Prevention

The findings discussed above suggest the intriguing possibility of prevention. Iarkov et al. discuss the cholinergic system as a therapeutic target of cotinine to prevent cognitive symptoms and dementia in Parkinson's disease (PD), characterized by the loss of dopaminergic neurons in the substantia nigra, resulting in progressive impairment of cognition and motor abilities. Nicotinic acetylcholinergic receptors (nAChR) are expressed in the striatum where their signals reduce neuroinflammation and facilitate neuronal survival, neurotransmitter release, and synaptic plasticity. Because of the deficit of nAChR in PD, inhibition of nAChR loss in the striatum may prevent dopaminergic neuron loss in the striatum. Nop et al. specifically examine the hypothetical benefits of using nicotine to improve cognition in non-demented healthy older adults. Studies in older adults with cognitive impairment show that chronic nicotine administration may benefit cognition in healthy older adults, depending on future evidence of the safety of nicotine dosing.

## Conclusion

Questions remain of how nicotine treatment in normal aging should proceed, including length of treatment, dose of nicotine, handling of smokers, effects of AD risk factors, and many others. While data from studies of psychiatric and memory-impaired subjects indicate that nicotine may relieve cognitive symptoms, it is mandatory to test the benefits of nicotine in normal aging in order to fill gaps in the literature and to verify the extent to which nicotine is useful as a pharmacologic agent that prevents pathological aging.

## Author contributions

The author confirms being the sole contributor of this work and has approved it for publication.
